# Critical role of high-dose Astragalus in Buyang Huanwu Decoction for enhancing neurovascular coupling in a Qi Deficiency and Blood Stasis animal model

**DOI:** 10.1186/s13020-026-01443-8

**Published:** 2026-06-18

**Authors:** Kaichao Hu, Juxiang Yang, Guining Wei, Yaxian Zhao, Shifeng Chu, Liqing Li, Shasha Wang, Shanhe Qu, Lixiang Chen, Yuxin Zhang, Xu Yan, Zhao Zhang, Naihong Chen, Wenbin He

**Affiliations:** 1 College of Pharmacy, Changchun University of Chinese Medicine, Changchun, 130012 Jilin China; 2 Shanxi Key Laboratory of Integrative Systemic Regulation for Qi-Blood Homeostasis, National International Joint Research Center for Molecular Chinese Medicine, Shanxi University of Chinese Medicine, Taiyuan, 030024 China; 3 State Key Laboratory of Bioactive Substances and Functions of Natural Medicines, Institute of Materia Medica, Chinese Academy of Medical Sciences & Peking Union Medical College, Beijing, 100050 China; 4 Guangxi Key Laboratory of Traditional Chinese Medicine Quality Standards, Guangxi Institute of Chinese Medicine & Pharmaceutical Science, Nanning, 530022 China

**Keywords:** Buyang Huanwu Decoction, High-dose Astragalus, Qi Deficiency and Blood Stasis Syndrome, Neurovascular unit, Neurovascular coupling

## Abstract

**Background:**

Buyang Huanwu Decoction (BHD) is a classical Traditional Chinese Medicine (TCM) formula effective for treating Chronic Cerebral Ischemia with Qi Deficiency and Blood Stasis Syndrome (CCI_QDBS_), which is known to alleviate neuronal damage and improve cerebral blood flow. The use of a high dose of Astragalus (Huangqi) has been clinically emphasized as crucial to BHD's efficacy, yet the pharmacological basis for this requirement remains unelucidated.

**Objective:**

This study aimed to compare the therapeutic efficacy of BHD with a high dose of Astragalus (120 g, BHD_HA_) versus a low dose (30 g, BHD_LA_) in treating CCI_QDBS_ and to investigate the underlying mechanisms.

**Methods:**

A mouse model of CCI_QDBS_ was established by combining systemic hypotension with bilateral common carotid artery occlusion and sleep deprivation. The necessity of high-dose Astragalus in BHD was evaluated by assessing syndrome-specific symptoms, cognitive performance, and brain functional connectivity via functional magnetic resonance imaging (fMRI). Neurovascular coupling (NVC) function was visualized in real-time using two-photon in vivo microscopy. RNA sequencing and immunofluorescence were employed to explore the molecular mechanisms by which BHD_HA_ improves NVC and mitigates brain injury. Finally, the role of nuclear Factor IA (NFIA) was validated through targeted gene silencing using an AAV-shRNA vector (pAVV-U6-sh*Nfia*).

**Results:**

BHD_HA_ demonstrated significantly superior therapeutic effects compared to BHD_LA_. It more effectively ameliorated CCI_QDBS_-induced deficits, including memory impairment and fatigue-like behaviors, and significantly enhanced brain functional connectivity. Mechanistically, BHD_HA_ induced the morphological recovery of astrocytes, increased their perivascular coverage, and restored impaired NVC function. These beneficial effects were found to be dependent on the upregulation of NFIA. Silencing *Nfia* abolished the therapeutic advantages of BHD_HA_ in improving NVC and cognitive function.

**Conclusion:**

Our findings demonstrate that the high dose of Astragalus is essential for the therapeutic efficacy of BHD under the conditions of the present experimental model. BHD_HA_ alleviates CCI_QDBS_ by modulating NFIA to maintain the stability of the neurovascular unit (NUV), thereby improving neurovascular coupling, remodeling brain functional connectivity, and restoring cognitive function. This study provides a scientific basis for the high-dose application of Astragalus in this classical formula. This study provides a scientific basis for the high-dose application of Astragalus in the treatment of CCI_QDBS_ using BHD.

**Supplementary Information:**

The online version contains supplementary material available at 10.1186/s13020-026-01443-8.

## Introduction

Chronic cerebral ischemia (CCI) is a prevalent form of ischemic injury defined by a sustained reduction in cerebral blood flow, often to a level of 25–45 mL/100 g/min, which represents a critical threshold between normal and necrotic brain tissue [8]. CCI can manifest as both a prodromal phase of acute ischemic stroke and a persistent pathological state during post-stroke recovery [3, 24]. Furthermore, CCI is a significant trigger for vascular cognitive impairment and dementia [9]. Therefore, promptly resolving the state of chronic ischemia is critical for the prevention and treatment of ischemic cerebrovascular diseases.

In TCM, CCI is often attributed to a syndrome of "Qi Deficiency and Blood Stasis (QDBS)". Qi is considered the fundamental force driving blood circulation. Consequently, the primary therapeutic strategy involves tonifying Qi and invigorating blood circulation. Buyang Huanwu Decoction (BHD), first recorded by Wang Qingren in "Yilin Gai Cuo" during the Qing Dynasty, is a cornerstone formula for treating this syndrome. BHD is composed of seven herbal ingredients: *Astragalus* (Huangqi), *Angelica sinensis* (Danggui), *Paeonia lactiflora* (Chishao), *Pheretima aspergillum* (Dilong), *Ligusticum chuanxiong* (Chuanxiong), *Carthamus tinctorius* (Honghua), and *Prunus persica* (Taoren). BHD is renowned for its ability to ameliorate post-stroke QDBS, with clinical observations describing it as enabling "paralyzed patients to walk again". A defining characteristic of this formula is the use of a high dose of Astragalus, typically 120 g, which is 20–40 times the dosage of the other herbs. However, the precise mechanism underlying the heavy reliance on Astragalus remains unreported. This has led to inconsistent clinical application, with Astragalus dosages in BHD varying from 30 to 120 g, resulting in variable efficacy that significantly hinders its clinical utility [19, 35].

Astragalus is a principal herb for "propelling Qi" (*Xing-Qi*) in TCM. The TCM theory that "Qi commands blood while nourishing its formation" exhibits a remarkable conceptual congruence with the modern physiological concept of neurovascular coupling (NVC). NVC dynamically modulates regional cerebral blood flow to match fluctuating energy consumption in activated brain areas. Long-term cerebral hypoperfusion-induced CCI commonly triggers NVC dysfunction [31]. Inefficient NVC deprives neurons of essential nutrients, leading to neuronal functional failure and exacerbating clinical symptoms such as memory impairment, cognitive decline, and psychomotor slowing [25]. The neurovascular unit (NVU) serves as the structural and functional basis for NVC. Therapeutic interventions targeting the NVU to enhance coupling efficacy are a promising strategy to dynamically adjust blood supply according to neuronal activity, thereby maintaining brain homeostasis and preventing the progression of cerebrovascular diseases [11]. This raises a compelling question of whether the traditional *Xing-Qi* effect of Astragalus linked, in modern pharmacological terms, to the regulation of neurovascular coupling.

This study aimed to verify the necessity of a high dose of Astragalus in BHD for treating QDBS syndrome. Animal models with QDBS were treated with high-dose Astragalus-containing BHD (BHD_HA_) and low-dose Astragalus-containing BHD (BHD_LA_), respectively. General physiological symptoms and cognitive behaviors were first evaluated to confirm the superiority of high-dose administration. Brain functional connectivity was analyzed via functional magnetic resonance imaging, and in vivo two-photon imaging was applied to achieve real-time visualization of NVC changes, so as to identify altered cerebral functions. RNA-sequencing was performed to screen differential signaling pathways and hub genes for mechanistic exploration. Gene silencing assays were finally conducted to verify the regulatory role of key genes in mediating the pharmacological effects of high-dose Astragalus. Collectively, this research clarifies the rationality and differential efficacy of high-dose Astragalus application in BHD, providing solid experimental evidence for standardized clinical medication and deeper mechanistic interpretation of this classic TCM formula.

This study aimed to verify the necessity of high-dose Radix Astragali in BHD against QDBS. Animal models with QDBS were treated with high-dose Astragalus-containing BHD (BHD_HA_) and low-dose Astragalus-containing BHD (BHD_LA_), respectively. General physiological symptoms and cognitive behaviors were first evaluated to confirm the superiority of high-dose administration. Brain functional connectivity was analyzed via functional magnetic resonance imaging, and in vivo two-photon imaging was applied to achieve real-time visualization of NVC changes, so as to identify altered cerebral functions. RNA-sequencing was performed to screen differential signaling pathways and hub genes for mechanistic exploration. Gene silencing assays were finally conducted to verify the regulatory role of key genes in mediating the pharmacological effects of high-dose Astragalus. Collectively, this research clarifies the scientific basis and differential efficacy of high-dose Astragalus application in BHD, providing solid experimental evidence for standardized clinical administration and deeper mechanistic interpretation of this classic TCM formula.

## Materials and methods

### Preparation of Buyang Huanwu Decoction (BHD)

The formulation of BHD was based on the original record in "Yilin Gai Cuo" (Correcting the Errors in the Forest of Medicine). The decoction included the following components: *Astragalus* (Huangqi) (Fisch. Lot: 24072601) at a high dose of 120 g or a low dose of 30 g, *Angelica sinensis* (Danggui) (Oliv. Lot: 24071208) 6 g, *Paeonia lactiflora* (Chishao) (Pall. Lot: 24071120) 4.5 g, *Pheretima aspergillum* (Dilong) (E.Perrier Lot: 24071036) 3 g, *Ligusticum chuanxiong* (Chuanxiong) (Hort. Lot: 24070802) 3 g, *Carthamus tinctorius* (Honghua) (L. Lot: 24071507) 3 g, and *Prunus persica* (Taoren) (L. Lot: 24070605) 3 g.

For preparation, the herbs were immersed in distilled water (5 times the volume of the herbs' weight, *v/w*) for 1 h. Subsequently, the mixture was boiled at 100 °C for 30 min, followed by simmering over low heat for 1 h. The resulting extract was filtered through three layers of gauze. This extraction process was repeated once with the remaining residue. The filtrates from both extractions were combined and then concentrated to yield BHD with a low dose of Astragalus (BHD_LA_), equivalent to 0.70 g of crude drug per mL, and BHD with a high dose of Astragalus (BHD_HA_), equivalent to 1.90 g of crude drug per mL. The daily dosage administered to mice was 0.1 mL per 10 g of body weight. This dosage was determined based on a 70 kg adult human equivalent, using a human-to-mouse dose conversion ratio of 1:9.

A validated HPLC method was established for simultaneous determination of 10 active compounds in BHD using an Agilent 1200 liquid chromatography system equipped with an Agilent Eclipse XDB-C18 column (250 × 4.6 mm, 5 μm). The flow rate was set at 1.0 mL/min. Detection was performed with a diode array detectorusing wavelength switching (207 or 240 or 300 nm) and an evaporative light scattering detector with the drift tube maintained at 60 °C and nitrogen carrier gas flow rate of 1.6 mL/min. The mobile phase consisted of 0.1% formic acid (A) and acetonitrile (B) with a gradient elution program. Samples were prepared by ultrasonic extraction with 70% methanol followed by centrifugation and filtration. This method was qualified for quality control and pharmacodynamic studies. See Supplementary Material Figure S1 for the chromatogram.

### Reagents and instruments

Ginaton (Dr. Willmar Schwabe GmbH & Co. KG); Isoflurane anaesthetic (RWD Life Science Co.,Ltd, R510-22-10); GFAP Antibody (Dako, M0761); NFIA Antibody (ABclonal, A3258); Donkey Anti-rabbit Alexa Fluor 594 (Invitrogen, A21207); Donkey Anti-Mouse Alexa Fluor 488 (Invitrogen, A 21202); pAAV-hSyn-jGCaMP7s-WPRE and pAAV-CMV-Luc2-WPRE-U6-shRNA(*Nfia*) adeno-associated virus (OBiO Technology); Agarose Gel (Invitrogen, 16520050); Dental Casting Material (Shanghai New Century Dental Materials Co., Ltd.); TRITC-Dextran 155 kDa (Sigma, T1287); KW-BD Sleep Deprivation Device (Nanjing Calvin Biotechnology Co., Ltd.); Animal Anesthesia Machine (MATRX); High-Resolution Camera (Guangzhou Bohan Electronics Co., Ltd.); Ultrasonic Cleaner (Shanghai Zhixin Instrument Co., Ltd.); Grip Testing Instrument (Jinan Yiyan Technology Development Co., Ltd.); PharmaScan 70/16 US MRI spectrometer (Bruker, Germany); Stereotaxic Injection Apparatus (RWD Life Science Co.,Ltd); Cytation C10 (Biotek); Freeze Dryer (Shanghai Licheng Technology Co., Ltd.); Two-Photon Microscope (Olympus, Japan).

### Animals and drug administration

SPF-grade male C57BL/6 J mice (6–8 weeks old, 22 ± 2 g) purchased from SPF (Beijing) Biotechnology Co., Ltd. were housed under controlled conditions (temperature: 24 ℃ ± 1 ℃; humidity: 55% ± 5%) with ad libitum accesss to standard chow and sterilized water. Twelve h prior to surgery, food was withheld, while water remained available. Utilizing a random number table, animals were allocated into five groups: Sham, Model, BHD_LA_, BHD_HA_, and Ginaton Group (2 mg/mL) [29]. Ginaton was dissolved in sterile water to prepare a 2 mg/mL solution. Drug-treated groups received intragastric administration of 0.1 mL/10 g once daily; Sham/Model groups were given the same volume of sterile water via the same route. All interventions commenced on the first day of BCCAO. The study was approved by the Ethics Committee of the Institute of Materia Medica, Chinese Academy of Medical Sciences (Ethical Approval No. IMM-N-25–0098) and complied with animal welfare regulations.

### Preparation of CCI_QDBS_ combined disease model

The combined chronic cerebral ischemia with Qi deficiency-blood stasis (CCI_QDBS_) model [34] was established in mice through bilateral common carotid artery occlusion with systemic hypotension (BCCAO) [23] and phased sleep deprivation. Animals underwent 7-day pre-BCCAO sleep deprivation followed by 28-day post-operative deprivation in a standardized apparatus, with daily 20-h cycles (12:00–08:00). This protocol integrated surgical ischemia induction with sustained metabolic stress to replicate both cerebrovascular and TCM syndrome pathophysiology.

### Tongue surface image acquisition

Mouse tongue surface images were acquired via high-definition camera at two time points: 14 days and 28 days post-BCCAO sleep deprivation. This dual-stage capture protocol generated paired image datasets for longitudinal analysis of each subject.

### Forelimb grip strength test

The forelimbs of the mice were placed on a grip strength testing apparatus. Once the mice grasped the apparatus, the tail was gently lifted to pull the mouse backwards horizontally at a constant speed. The reading on the grip strength tester was recorded, completing one test. Each mouse underwent three tests, with a 15 min interval between each test.

### Open field test

The open field box was constructed with opaque black walls measuring 50 cm × 50 cm × 50 cm, and the white bottom was divided into 4 × 4 squares. The environment was kept quiet during the experiment to minimize disturbances. One day before modeling, the animals were individually placed in the open field box for 5 min to adapt. After adaptation, the box was wiped with 10% alcohol to remove odors. Following the modelling, the animals were placed in the central square, and the Smart 3.0 behavioral data collection software was used to record the total distance moved and the number of crossings within 5 min for each group of mice. After testing each mouse, the box was cleaned before proceeding with subsequent experiments.

### Barnes maze test

Behavioral data were recorded using Smart 3.0 software during a 4-day Barnes maze test. The apparatus consisted of a white metal platform (diameter: 120 cm, height: 50 cm) with 20 peripheral holes, one containing the target box. Daily sessions began with 5 min target box acclimation. Animals were then centrally placed after 3 min dark box confinement, with latency to enter the target hole recorded using a 5 min cutoff (300 s if unsuccessful). Post-trial procedures included repeated acclimation and ethanol cleaning to eliminate odor cues.

### Magnetic resonance imaging data collection and analysis

Functional MRI data analysis. At the beginning of each MRI examination, FieldMap and continuous local shimming were used to improve the homogeneity of the magnetic field. Subsequently, a T2-weighted TurboRARE sequence was used as an anatomical reference scan, followed by the acquisition of functional MRI using a free induction decay echo-planar imaging (FID-EPI) sequence, repeated 300 times. Resting-state fMRI data were acquired via FID-EPI (TR = 2000 ms, TE = 15 ms, 300 volumes, 40 slices, 0.35 mm thickness). Brain functional data sets were extracted, and the original data was converted to NIfTI format files using dcm2nii. Voxel size was increased by 10 times, and slice timing correction was performed with the middle layer as the reference layer. Realignment was conducted to align each subject's time-processed volume to the mean volume to eliminate any head motion. An average image was created from the 300 realigned volumes. After head motion correction, all subject data was registered to standard space using a two-step registration method with the ANTS (1.9.2) toolkit. Functional images were registered to the Allen Mice Brain using T2 and mean function images. Gaussian smoothing (smoothing kernel = 6 mm) and filtering (range 0.01–0.08 Hz) were then performed on the registered data using SPM12 and rest plus V1.2.8–130615 toolkits. Subsequently, regression covariate analysis and detrend analysis were performed to obtain the data for analysis. Functional connectivity between regions of interest (ROIs) was analyzed, Brain regions related to memory, somatosensory-motor were selected, including: CA1, CA3, DG-mo, DG-sg, FC, ccs, PL1, ORBm, BLAv, CP, SSp-bfd. The correlation coefficients between seed points were calculated and then Fisher-z normalized to obtain zFC.

### Stereotaxic viral injection

All stereotaxic surgeries were performed under isoflurane anesthesia (2% in O₂) with the animal's head fixed in a stereotaxic apparatus. After a midline scalp incision to locate bregma, viral vectors were injected. For monitoring neural activity, pAAV-hSyn-jGCaMP7s-WPRE was delivered unilaterally into the barrel cortex (AP: −3.52 mm, ML: + 1.5 mm, DV: −1.0 mm). The infusion proceeded over 10 min, followed by a 5 min needle retention period. This specific region was selected as it provides a robust and classic anatomical target for sensory-evoked NVC evaluation.

To achieve NFIA knockdown, pAAV-CMV-Luc2-WPRE-U6-shRNA (*Nfia*) was bilaterally injected into the lateral ventricle (AP: + 0.40 mm, ML: + 1.00 mm). This site was targeted to effectively reach the adjacent subventricular zone (SVZ), the primary origin of reactive astrocytes, thereby facilitating the investigation of NFIA-mediated astrogenesis [17]. At each injection tract, 100 nL of the virus was delivered to six different ventral depths (−3.40, −3.20, −3.00, −2.80, −2.60, and −2.40 mm).

### Surgical preparation and two-photon imaging

To monitor neuronal activity, mice first received stereotactic viral injections. Under isoflurane anesthesia, 1 μL of the adeno-associated virus pAAV-hSyn-jGCaMP7s-WPRE (titer: 8.61 × 10^12^ vg/mL) was injected into the barrel cortex. Three weeks post-viral expression, mice were anesthetized and a 3 mm cranial window was created over the barrel cortex, centered on the previous viral injection site. Briefly, after a midline scalp incision, a circular craniotomy was drilled and the bone flap was removed. The exposed brain was covered with 1% agarose and sealed with a 3 mm glass coverslip using cyanoacrylate adhesive. A titanium headplate was then cemented to the skull to allow for stable head fixation. Whisker stimulation was delivered using a custom-built, motor-driven device. The apparatus was centered around a GA12-N20 geared DC motor, regulated by a forward-reverse stop module to control the stimulation pattern. The system was powered by a portable battery pack and operated remotely to minimize experimental interference.

For imaging sessions, awake, head-fixed mice were positioned under an Olympus FVMPE-RS two-photon microscope, which was enclosed in a light-tight shield. Approximately 15 min prior to imaging, the vasculature was labeled via a tail-vein injection of 0.1 mL TRITC-Dextran (155 kDa, prepared at 10 mg/mL). Two-photon excitation was generated using a femtosecond laser (Mai Tai, Spectra-Physics) tuned to an excitation wavelength of 930 nm. Images were acquired through a 25 × water-immersion objective (Olympus, NA 1.05) with the acquisition controlled by FV10-ASW software. To validate the methodological synchrony of NVC, time-series images (320 × 320 pixels) were acquired at 4 µs per pixel to monitor changes in neuronal calcium signals and vascular dynamics simultaneously. Trial consisted of 200 consecutive frames to capture the response before, during, and after stimulation. To quantitatively assess blood flow parameters across groups, line-scans (XT mode) were performed along the central axis of target vessels, at 2 µs per pixel.

### RNA sequencing and analysis

Total RNA were extracted from brain tissue, and library construction was performed by Beijing Qinglian Biotech Co., Ltd. using the Illumina TruSeq Stranded mRNA LT Kit. The prepared libraries were sequenced on a NovaSeq 6000 platform (PE150). After trimming adapters and low-quality bases, clean reads were aligned to the reference genome. Gene expression was quantified, and differentially expressed genes (DEGs) were identified using an adjusted *p*-value threshold of < 0.05. The functional roles of DEGs were investigated through GO and pathway enrichment analysis.

### Immunofluorescence staining

At the experimental endpoint, mice were deeply anesthetized with isoflurane and transcardially perfused with PBS followed by 4% PFA. Brains were extracted, post-fixed, and sectioned into 50 µm coronal slices. For staining, free-floating sections underwent heat-induced antigen retrieval, were permeabilized with Triton X-100, and blocked with 8% serum solution. Sections were then incubated with primary antibodies overnight at 4 °C, followed by a 2-h incubation at room temperature with corresponding secondary antibodies. All incubation steps were flanked by thorough washes in PBS. Finally, sections were mounted with an anti-fade medium and imaged on a Cytation C10 confocal microscope.

### Statistical analysis

All data are expressed as mean ± SEM. GraphPad Prism 8.0.2 was used for data analysis. One-way ANOVA, followed by Tukey's *post-hoc* test, was applied to compare multiple groups. A *p*-value of < 0.05 was considered statistically significant.

## Results

### BHD_HA_ ameliorates QDBS syndrome in CCI_QDBS_ model

**Fig. 1:**
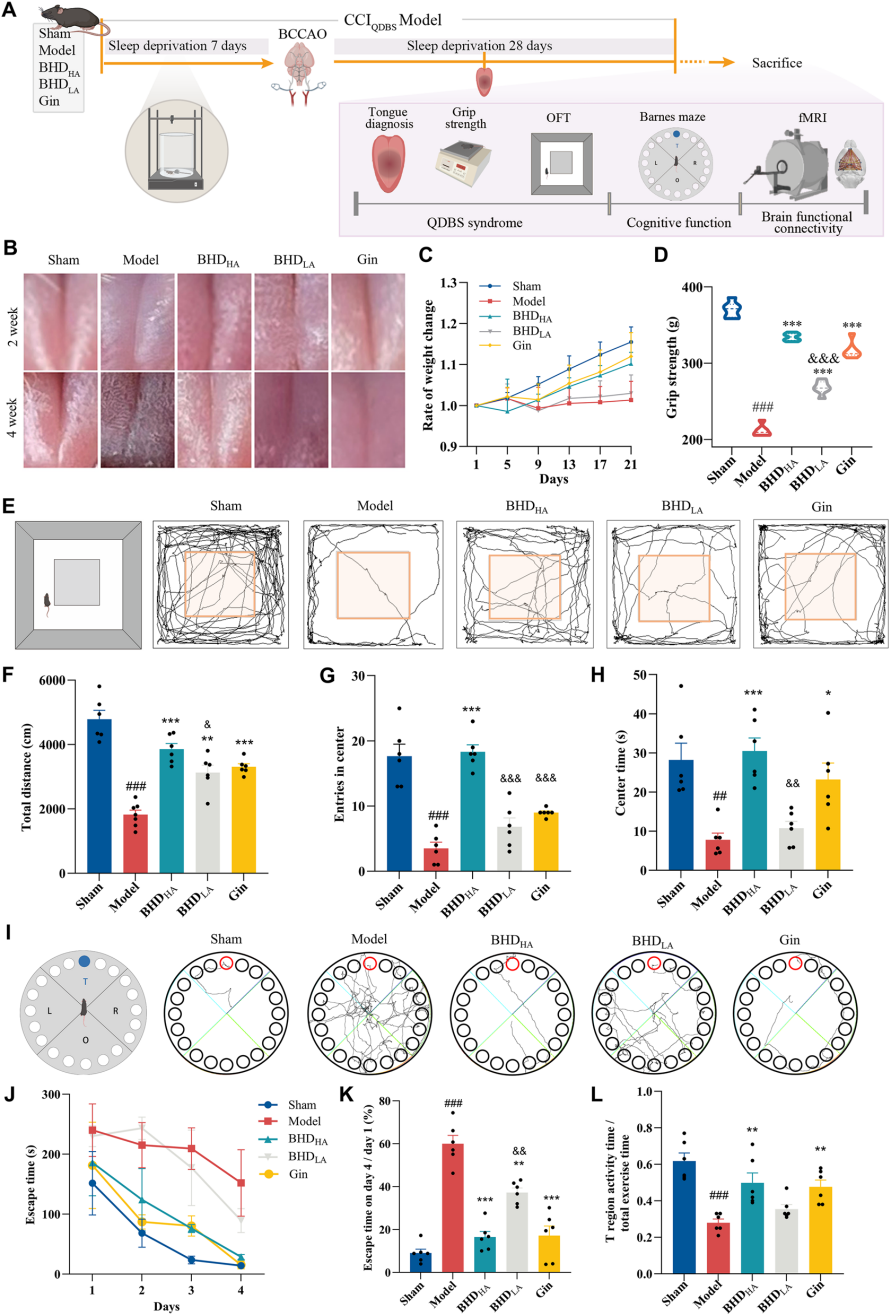
Effects of BHD_HA_ and BHD_LA_ on CCI_QDBS_-associated phenotypes. **A** Schematic of the experimental procedure. **B** Representative tongue images at 2 and 4 weeks post-BCCAO surgery. **C** Mouse body weight changes post-BCCAO surgery. **D** Forelimb grip strength test post-CCI_QDBS_. **E**–**H** OFT post-CCI_QDBS_: **E** OFT schematic and representative trajectories; **F** Total distance traveled; **G** Number of entries into the center zone; **H** Time spent in the center zone. **I**–**L** Barnes maze experiment. T, target quadrant; O, opposite quadrant; R, right adjacent quadrant; L, left adjacent quadrant.: **I** Maze schematic and representative movement trajectories; **J** Escape time of mice in each group; **K** Change in escape latency post-training (ratio of escape latency on day 4 to day 1); **L** Proportion of effective exploratory activity in the target quadrant on day 4 (exploration time/total time in target quadrant). Data are presented as mean ± SEM (n = 6). ^*##*^*p* < 0.01, ^*###*^*p* < 0.001 *vs.* Sham; ^*^p < 0.05, ^**^p < 0.01, ^***^p < 0.001 *vs.* Model; ^*&*^*p* < 0.05, ^*&&*^*p* < 0.01, ^*&&&*^*p* < 0.001 *vs.* BHD_HA_

QDBS syndrome is clinically characterized by a dark tongue with a white coating [18], weakness, lassitude, and impaired memory. Compared to the healthy tongues of sham-operated mice, model mice developed a characteristic dark, coated tongue, a primary sign of QDBS. This was accompanied by impaired weight gain and a significant reduction in grip strength (Fig. 1B–D). Treatment with BHD_HA_ markedly reversed these deficits, restoring normal tongue color, promoting weight gain, and significantly improving grip strength. The therapeutic effects of BHD_LA_ and Ginaton (Gin) were less pronounced. Notably, the grip strength in the BHD_HA_ group was approximately 23% greater than in the BHD_LA_ group (*p* < 0.001, Fig. 1D). In the open field test, model mice exhibited signs of fatigue and lassitude, showing significantly less total movement and reduced exploration of the center area compared to sham controls (Fig. 1E–H). Mice in the BHD_HA_ group showed significantly increased spontaneous activity (*p* < 0.001). In contrast, the improvements observed in the BHD_LA_ group were modest and significantly lower than in the BHD_HA_ group (*p* < 0.001 in Fig. 1G *p*< 0.01 in Fig. 1H). We then assessed cognitive function using the Barnes maze (Fig. 1I, J). Model mice displayed significant learning and memory impairments. BHD_HA_ treatment led to an improvement in learning, as demonstrated by a significantly shorter escape latency ratio compared to both the model (*p* < 0.001) and BHD_LA_ groups (*p* < 0.01) (Fig. 1K). Furthermore, on day 4, BHD_HA_-treated mice spent significantly more time exploring the target hole than Model mice (*p* < 0.01, Fig. 1L). This effect was not observed in the BHD_LA_ group. Taken together, these results demonstrate that BHD_HA_ effectively alleviates the behavioral and cognitive impairments in CCI_QDBS_ model, showing a therapeutic efficacy markedly superior to that of BHD_LA_ and Ginaton.

### BHD_HA_ effectively improves sensorimotor and cognitive brain functions impaired by CCI_QDBS_

**Fig. 2:**
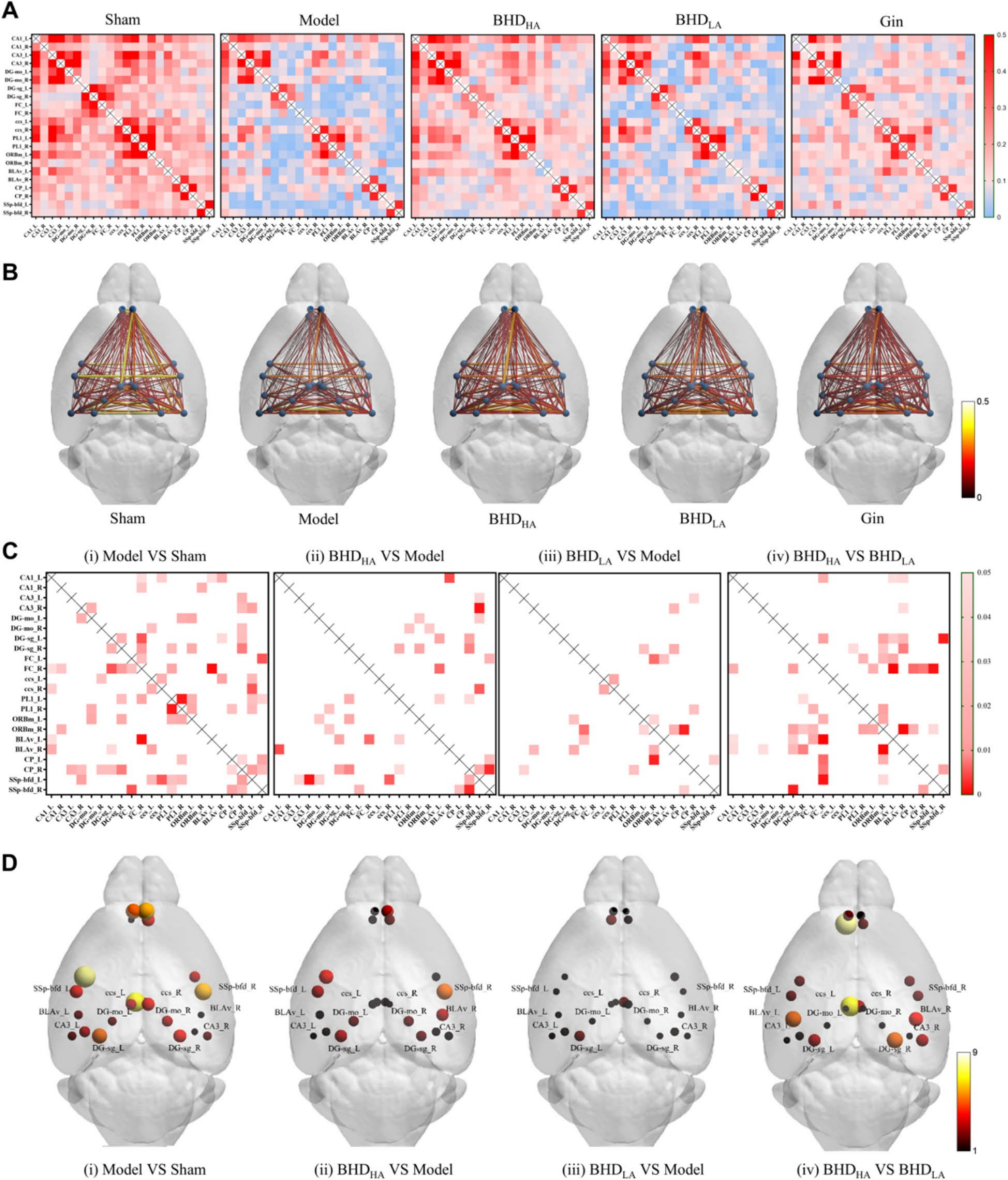
BHD_HA_ enhance functional brain connectivity post-CCI_QDBS_. **A** Heatmap illustrating the Z-scores of functional connectivity between ROIs for each group. **B** BrainNet Viewer visualization of functional brain connectivity. Edge thickness and color correspond to the Z-values, indicating the strength of functional connectivity. **C** P-value matrix highlighting significant differences in ROI functional connectivity (two-sample t-test). Color intensity indicates higher statistical significance. **D** Visualization of P-values. The size and color of the spheres represent the significantly altered connections. n = 5 per group

QDBS syndrome leads to sluggish thinking and cognitive decline. We utilized fMRI to examine neuronal activity and functional connectivity in brain regions associated with learning and memory. ROIs pertinent to CCI injury were selected, encompassing memory-related areas, the gustatory cortex, and somatosensory and motor brain regions, to quantify the strength of functional connectivity between them [4, 12, 15, 16]. The results indicated that brain functional connectivity in the CCI_QDBS_ Model group was significantly weakened. Conversely, the BHD_HA_ group exhibited enhanced functional connectivity, reflected by an increased Z-score (Fig. 2A). The heatmap visualization further revealed that the functional connectivity pattern in the BHD_LA_ group more closely resembled that of the Model group, whereas the BHD_HA_ group's pattern showed greater similarity to the Sham group. For intuitive visualization, BrainNet Viewer was employed (Fig. 2B), which showed that connections appearing yellow and thicker (indicative of stronger functional links) were more prominent between multiple brain regions in the BHD_HA_ group compared to the Model group. To highlight specific regional differences, P-value matrices were utilized for further analysis (Fig. 2C). We performed the following comparisons: (i) Model vs. Sham, (ii) BHD_HA_ vs. Model, (iii) BHD_LA_ vs. Model, and importantly, (iv) BHD_HA_ vs. BHD_LA_. We found that functional connectivity involving regions such as CA3, DG, ccs, BLAv, and SSp-bfd was significantly stronger in the BHD_HA_ group compared to both the Model and BHD_LA_ groups (Fig. 2D). This enhanced functional connectivity in the brain provides a compelling explanation for the behavioral improvements observed with BHD_HA_ treatment (as detailed in Fig. 1), thereby confirming that BHD_HA_ can effectively promote the recovery of sensorimotor and higher-order cognitive brain functions compromised by CCI_QDBS_.

### BHD_HA_ treatment restores cerebral hemodynamics in CCI_QDBS_

**Fig. 3:**
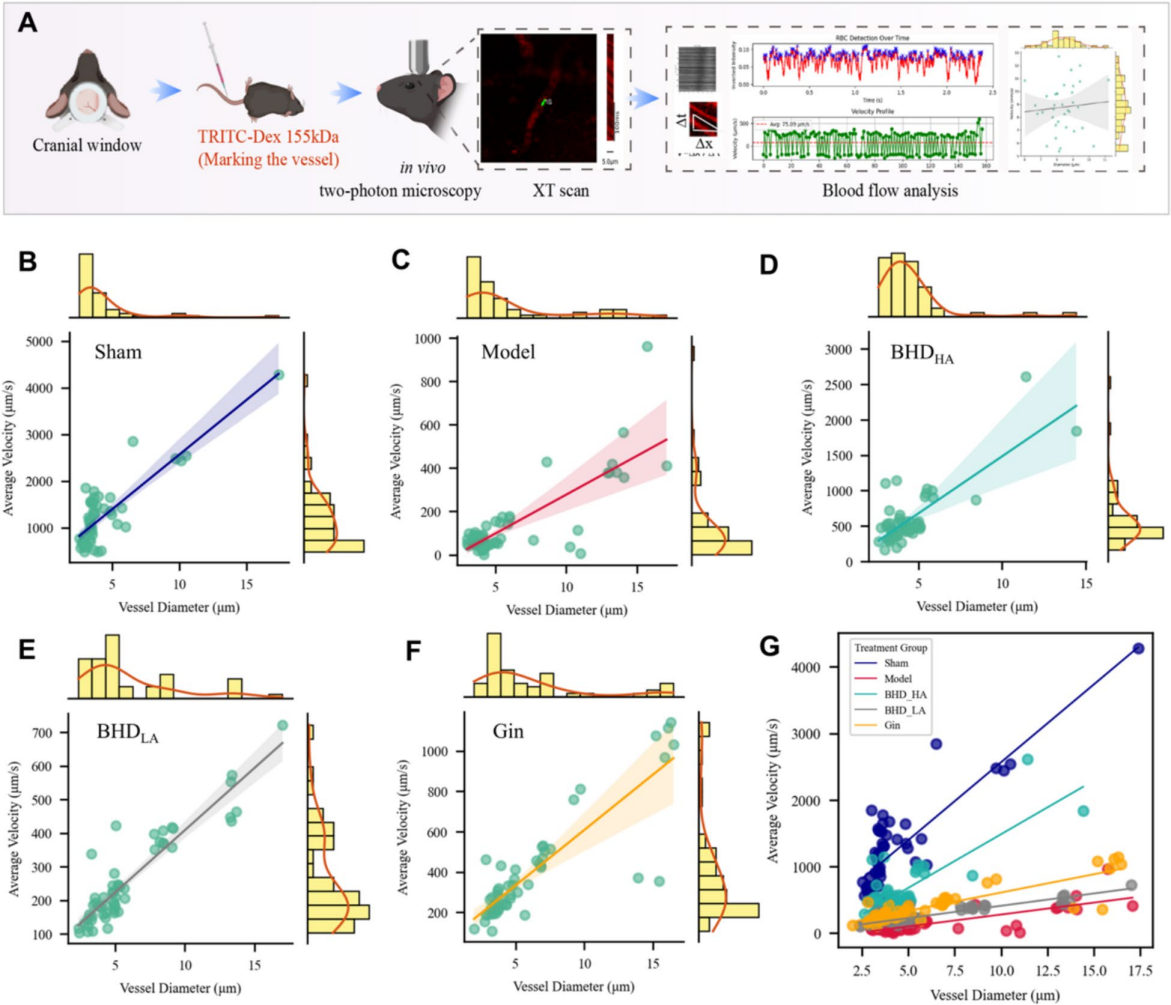
BHD_HA_ restores cerebral blood flow and vascular reactivity. **A** Schematic of the experimental workflow for analyzing cerebral hemodynamics using in vivo two-photon microscopy. The process includes cranial window preparation, intravenous injection of TRITC-Dextran to label plasma, acquisition of XT line-scan images from cortical vessels, and subsequent blood flow analysis. **B**–**F** Scatter plots of average blood flow velocity versus vessel diameter for individual capillaries in the **B** Sham, **C** Model, **D** BHD_HA_, **E** BHD_LA_, and **F** Gin treatment groups. Each point represents a single vessel measurement. The solid line indicates the linear fit of the velocity-diameter relationship, with the shaded area representing the 95% confidence interval. Histograms show the distribution of vessel diameters (top) and average velocities (right). **G** A composite plot of the linear fitting lines from all treatment groups, illustrating the comparative effects on vascular reactivity. The slope of each line indicates the efficiency of blood flow in relation to vessel diameter. n = 5 mice/group, N = 54 vessels for Sham, BHD_HA_, BHD_LA_, and Gin groups, N = 59 vessels for Model group. All 275 vessels analyzed

Impaired cerebral hemodynamics is a core pathological feature of CCI_QDBS_ syndrome and is fundamental for supplying nutrients to support brain activity. To investigate the overall hemodynamic state, we first labeled the blood plasma with TRITC-Dextran (155 kDa) and performed in vivo two-photon microscopy, using XT line scans to analyze blood flow (Fig. 3A). We plotted the relationship between blood flow velocity and vessel diameter for each group, and compared overall blood flow velocity among groups using the slope of the linear fits. As shown in Fig. 3C and Fig. 3G, compared with the sham group, the model group exhibited a much flatter slope for the relationship between vessel diameter and blood flow velocity, indicating slow blood flow and confirming the successful establishment of the CCI_QDBS_ model. In contrast, both the scatter plot data and the hemodynamic fitting line for the BHD_HA_ group (Fig. 3D) were substantially elevated compared to the Model group. This demonstrates that for the same vessel diameter, blood flow velocity was significantly higher following BHD_HA_ treatment. Furthermore, the steep slope of the BHD_HA_ regression line closely resembled that of the healthy Sham group (Fig. 3B), indicating a robust restoration of vascular function. Notably, the therapeutic effect of BHD_HA_ was more effective than that of BHD_LA_ in Fig. 3E, as summarized by the comparison of all treatment groups in Fig. 3G.

### BHD_HA_ improves NVC in CCI_QDBS_ mice

**Fig. 4:**
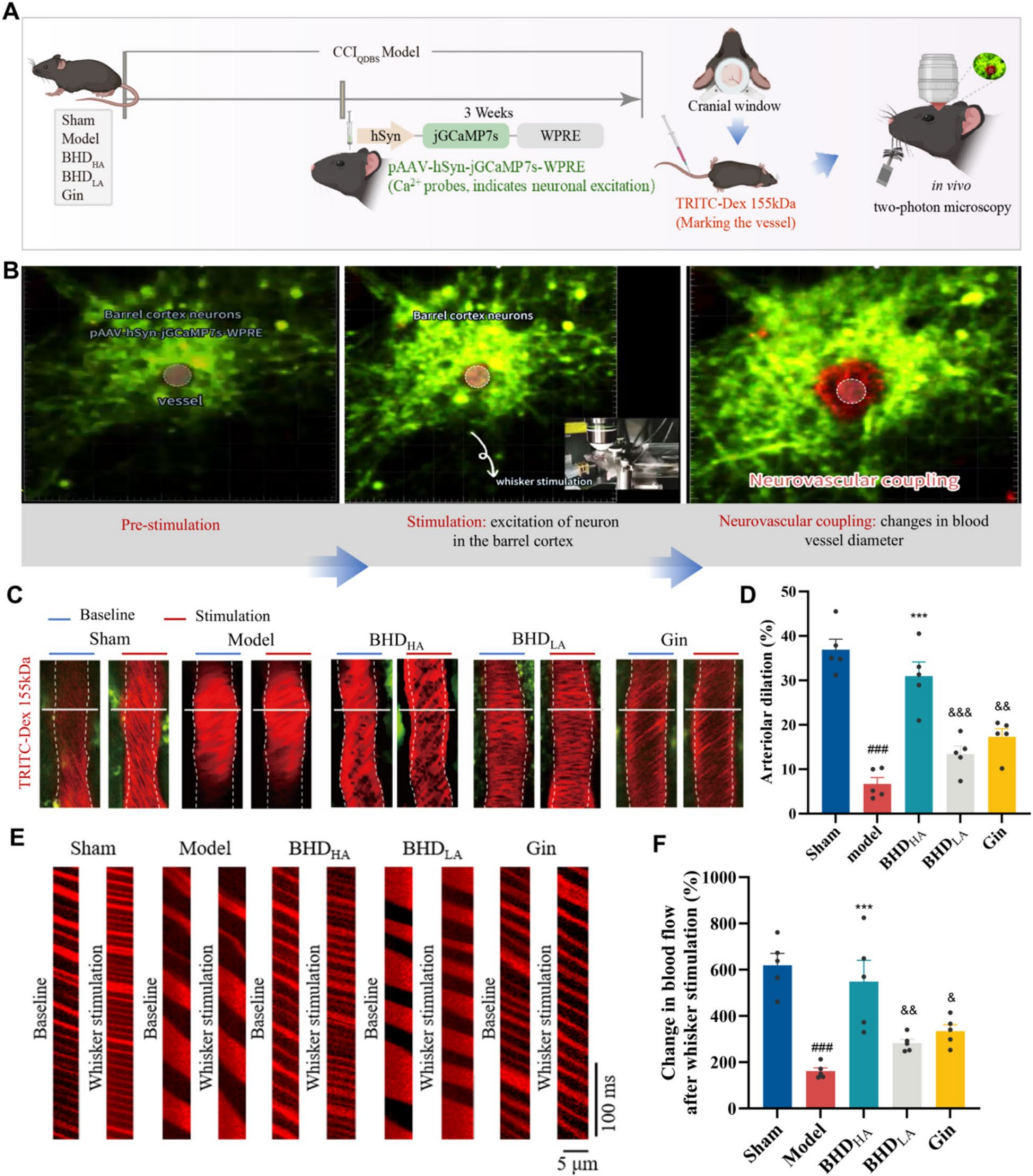
In vivo assessment of NVC function. **A**, **B** Schematic of in vivo NVC assessment using two-photon microscopy. Experimental setup illustrating stereotactic injection of jGCaMP7s in the barrel cortex for neuronal activity, cranial window preparation, TRITC-Dextran for vascular imaging, and whisker stimulation to monitor neurovascular dynamics. **C** Representative images illustrating microvascular diameter before and after whisker stimulation. **D** Dilation analysis following whisker stimulation. **E** Representative images of XT line-scan. **F** Percentage change in microvascular blood flow following whisker stimulation. Data are presented as mean ± SEM (n = 5). ^*##*^*p* < 0.01, ^*###*^*p* < 0.001 *vs.* Sham; ^***^p < 0.001 *vs.* Model; ^*&*^*p* < 0.05, ^*&&*^*p* < 0.01, ^*&&&*^*p* < 0.001 *vs.* BHD_HA_

We next sought to determine if BHD_HA_ could restore functional neurovascular coupling (NVC), the critical mechanism responsible for matching local blood flow to the energy demands of neuronal activity [1]. To this end, we utilized in vivo two-photon microscopy to measure stimulus-evoked vascular responses in the barrel cortex, which were induced by whisker stimulation, a robust and well-characterized sensory paradigm [7, 38]. As illustrated in Fig. 4A, pAAV-hSyn-jGCaMP7s-WPRE was stereotactically injected into the barrel cortex of mice to label neuronal activity. Three weeks post-viral expression, a cranial window was prepared, and TRITC-Dextran (155 kDa) was administered via tail vein injection to delineate vascular morphology and enable blood flow velocity assessment. A custom-built whisker stimulator delivered controlled stimulation to barrel cortex neurons, while the ensuing effects on neuronal activity (indicated by jGCaMP7s) and vascular dynamics (indicated by TRITC-Dextran) were monitored in real-time using two-photon microscopy (Fig. 4A, B). Movie S1 visually demonstrates that whisker stimulation-induced barrel cortex excitation leads to a synchronous increase in vascular diameter, accompanied by an increase in blood flow velocity and volume, thus allowing for the direct visualization of NVC. Based on these NVC assessments, we observed distinct differences in microvascular diameter changes following whisker stimulation. In the Sham group, the vessel diameter increased by approximately 36.88% post-stimulation, whereas in the Model group, the increase was only about 6.68%. The BHD_HA_ group exhibited a marked increase in vessel diameter, approximately double that observed in the BHD_LA_ group (*p* < 0.001) (Fig. 4C, D). These findings indicate that NVC function was severely impaired in the Model group, and BHD_HA_ was significantly more effective than BHD_LA_ in preserving this crucial coupling mechanism. Furthermore, we analyzed changes of blood flow of microvessels in each group using XT line-scans (Fig. 4E). Analysis of blood flow velocity before and after stimulation (Fig. 4E), and the corresponding change in blood flow (Fig. 4F), clearly demonstrated that the cerebral vasculature in the BHD_HA_ group could rapidly respond to changes in neuronal activity by increasing blood flow perfusion, indicating a more significant improvement in NVC function. This observation aligns with the findings on enhanced brain functional connectivity. This study visually demonstrated in vivo that BHD_HA_ improves NVC function in CCI_QDBS_ mice, and this therapeutic advantage appears to be associated with the high-dose Astragalus component within the BHD_HA_ formulation.

### BHD_HA_ reshapes the brain transcriptome in CCI_QDBS_ mice

**Fig. 5:**
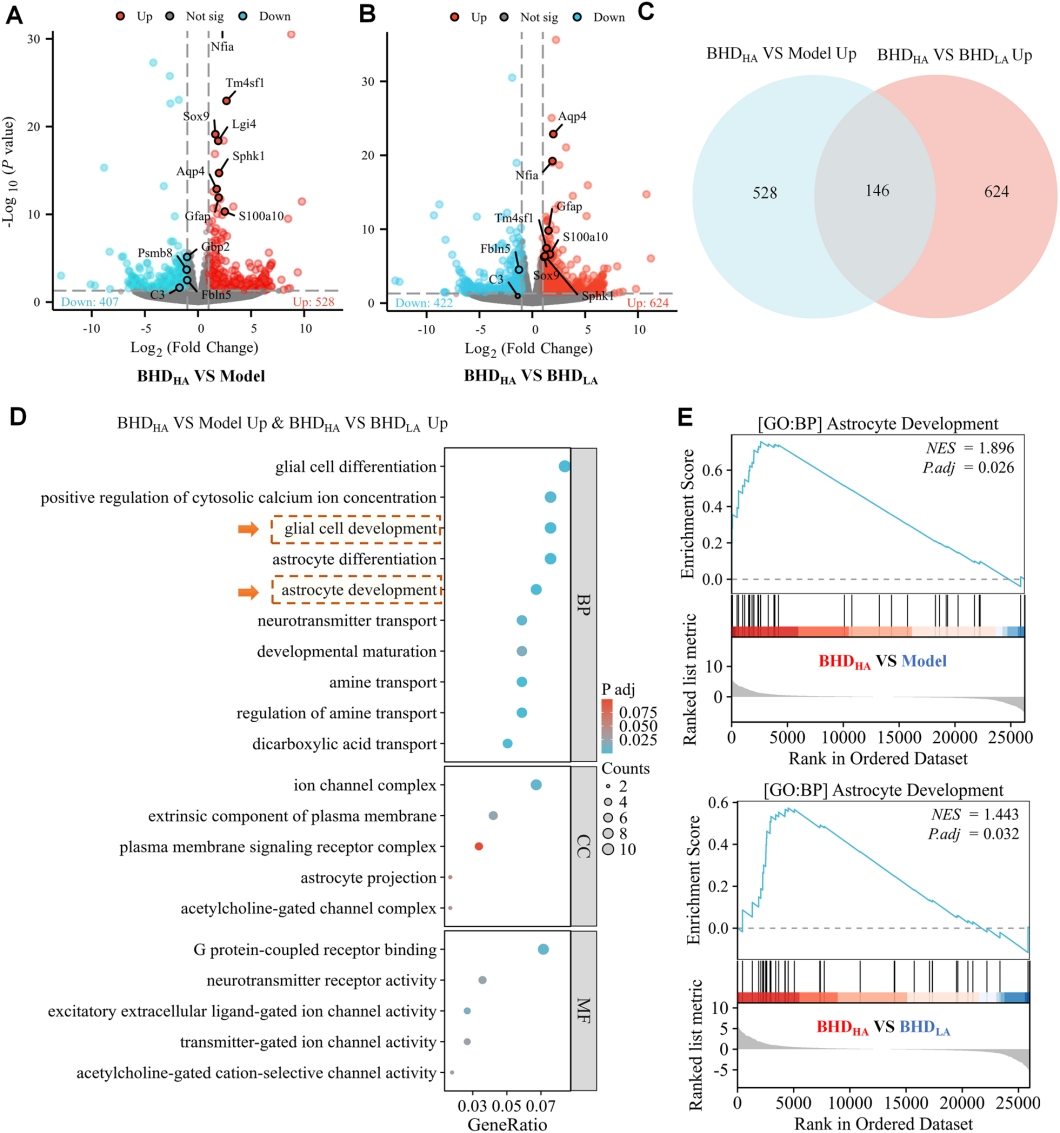
Gene expression analysis of BHD treatment in CCI_QDBS_ mice. **A** Volcano plot analysis of DEGs between BHD_HA_ and model groups. **B** Volcano plot analysis of DEGs between BHD_HA_ and BHD_LA_ groups. **C** Venn diagram analysis of DEGs. **D** Gene Ontology biological process enrichment of Up-regulated DEGs (BHD_HA_ VS Model Up & BHD_HA_ VS BHD_LA_ Up). Enrichment is evident in astrocyte development. **E** GSEA revealed upregulated "astrocyte development" in the BHD_HA_ group. n = 3

To elucidate the mechanism by which BHD_HA_ improves NVC in CCI_QDBS_ mice, we conducted a whole-genome RNA sequencing analysis. Using filtering criteria of *P* < 0.05 and FC > 2, a total of 935 differentially expressed genes were identified between the Model and BHD_HA_ groups (Fig. 5A), and 1046 differentially expressed genes were found between the BHD_HA_ and BHD_LA_ groups (Fig. 5B). A total of 146 genes were co-upregulated in the BHD_HA_ group compared to the Model and BHD_LA_ groups (Fig. 5C). GO enrichment analysis of these commonly upregulated genes demonstrated that biological processes such as "astrocyte development", and "glial cell proliferation" were significantly enriched after BHD_HA_ treatment (Fig. 5D). Moreover, gene set enrichment analysis (GSEA) revealed upregulated "astrocyte development" in the BHD_HA_ group compared to the Model and BHD_LA_ groups, with enrichment scores of 1.896 and 1.443 respectively (Fig. 5E). It has been reported that astrocytes, as a component of the neurovascular unit, interact with the vascular system and serve as a communication bridge between neurons and blood vessels, playing a regulatory role in capillaries and blood flow, making them an important component of NVC [30]. This suggests that the effect of BHD_HA_ in improving NVC function in CCI_QDBS_ mice may be related to the regulation of astrocyte development.

### BHD_HA_ restores astrocytic vascular coverage in CCI_QDBS_ mice

**Fig. 6:**
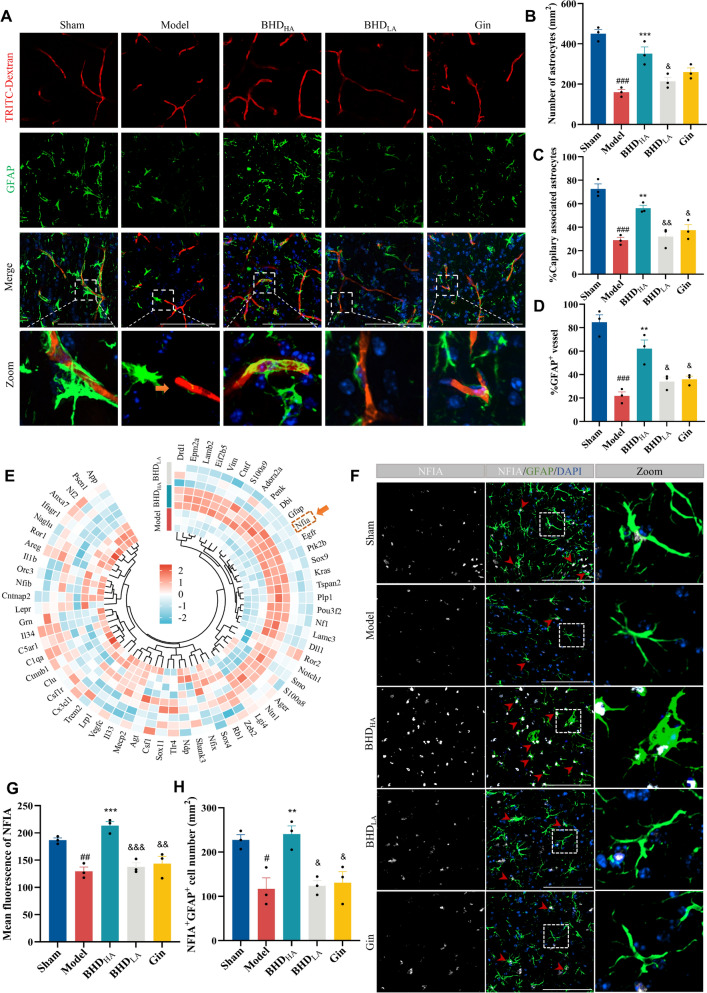
BHD_HA_ promotes astrocyte development and enhances vascular coverage in CCI_QDBS_ mice. **A** Representative immunofluorescence images of astrocytes (GFAP, green) and blood vessels (TRITC-Dextran, red) in the cortex of mice, bar = 100 μm; **B** Quantification of the total number of astrocytes; **C** Percentage of cerebrovascular-associated astrocytes; **D** Percentage of blood vessels covered with astrocytes; **E** Heatmap illustrating the expression levels of genes related to glial cell development in the Model, BHD_HA,_ and BHD_LA_ groups; **F** Representative immunofluorescence images of astrocytic NFIA in the cortex, bar = 100 μm; **G**) Quantification of the mean fluorescence intensity of NFIA; **H** Quantification of the number of NFIA^+^/GFAP^+^ cells, Data are presented as mean ± SEM. n = 3. ^*##*^*p* < 0.01, ^*###*^*p* < 0.001 *vs.* Sham; ^**^p < 0.01, ^***^p < 0.001 *vs.* Model; ^*&*^*p* < 0.05, ^*&&*^*p* < 0.01, ^*&&&*^*p* < 0.001 *vs.* BHD_HA_

To determine if the BHD_HA_-mediated improvement in NVC function was linked to astrocytes, we quantified the total astrocyte population, the proportion of cerebrovascular-associated astrocytes (CAAs), and the extent of astrocytic vascular coverage. The CCI_QDBS_ model induced a significant loss of astrocytes and reduced vascular coverage compared to controls. Treatment with BHD_HA_ markedly reversed these deficits, proving significantly more effective than BHD_LA_ (*p* < 0.05 in Fig. 6B, D; *p* < 0.01 in Fig. 6C). Moreover, BHD_HA_ was superior to Ginaton in restoring both the CAA population and astrocytic vascular coverage (*p* < 0.05 for both). These findings indicate that BHD_HA_ not only increases the overall number of astrocytes but also enhances their functional association with the vasculature.

To further explore the mechanism by which BHD_HA_ affects astrocyte development, we investigated key regulatory genes from our RNA-seq data. This analysis revealed that *NFIA*, a transcription factor known to drive astrogenesis [6], was significantly upregulated in the BHD_HA_ group compared to both the model and BHD_LA_ groups (Fig. 6E). We validated this finding at the protein level via immunofluorescence (Fig. 6F). This confirmed that NFIA protein levels were significantly reduced in the cortices of model mice (*p* < 0.01). In contrast, BHD_HA_ treatment robustly increased both the fluorescence intensity of NFIA and the number of NFIA-positive astrocytes, obviously outperforming the model and BHD_LA_ groups (*p* < 0.001 and *p* < 0.01, respectively; Fig. 6G, H). Additionally, our transcriptomic analysis showed an upregulation of genes associated with the neuroprotective A2-type astrocyte phenotype in the BHD_HA_ group (Fig. S2). Collectively, these results suggest that BHD_HA_ promotes the generation of functional astrocytes by upregulating the transcription factor NFIA, thereby helping to restore the integrity of the neurovascular unit.

### BHD_HA_ restores NVC function by upregulating NFIA

**Fig. 7:**
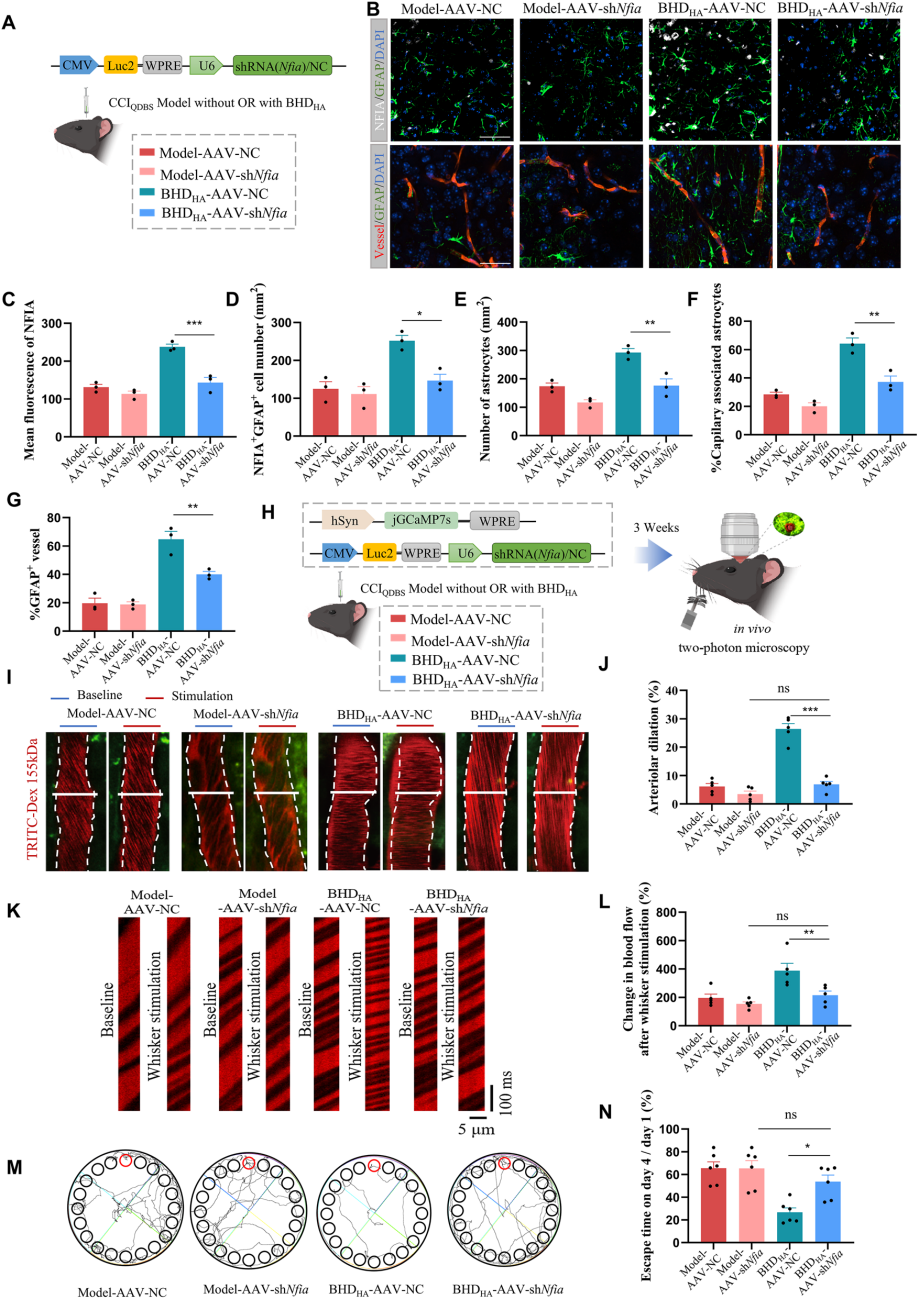
NFIA knockdown reverses the beneficial effects of BHD_HA_ on NVC and spatial memory. **A** Schematic diagram of AAV mediated *Nfia* knockdown. **B** confocal images of cortical sections stained for NFIA, the astrocyte marker GFAP, and blood vessels (TRITC-Dextran 155), bar = 50 μm. **C** Quantification of NFIA mean fluorescence intensity. **D** Quantification of the number of NFIA^+^/GFAP^+^ cells. **E** Quantification of the total number of astrocytes; **F** Percentage of CAAs. **G** Percentage of blood vessels covered with astrocytes. n = 3. **H** Schematic diagram of NVC test using two-photon microscopy. **I** Representative images illustrating microvascular diameter before and after whisker stimulation. **J** Dilation analysis following whisker stimulation. **K** Representative images of XT line-scan. **L** Percentage change in microvascular blood flow following whisker stimulation. n = 5. **M** Representative movement trajectories of Barnes maze. **N** Proportion of effective exploratory activity in the target quadrant on day 4 (exploration time/total time in target quadrant). n = 6. ^*^p < 0.05, ^**^p < 0.01, ^***^p < 0.001

To directly investigate the role of NFIA in BHD_HA_-mediated improvement of NVC function, we constructed an adeno-associated virus (pAAV-sh*Nfia*) targeting Nfia. Since the subventricular zone (SVZ) is the primary source of reactive astrocytes that migrate to ischemic lesions after injury, we performed multi-point injections of pAAV-U6-sh*Nfia* (or a negative control, pAAV-U6-shNC) into the brain tissue surrounding the lateral ventricle to achieve this knockdown (Fig. 7A). Results showed that compared to the BHD_HA_-AAV-shNC group, the BHD_HA_-AAV-sh*Nfia* group exhibited significantly reduced NFIA expression (*p* < 0.001) and decreased numbers of NFIA^+^ astrocytes (*p* < 0.05) (Fig. 7B–D). Additionally, total number of astrocytes (Fig. 7E *p* < 0.01), the percentage of CAAs (Fig. 7F *p*< 0.01), and the coverage of blood vessels by astrocytes (Fig. 7G *p*< 0.01) were markedly diminished, indicating that *Nfia* knockdown in the SVZ suppressed BHD_HA_'s ability to promote astrocyte development. Furthermore, in BHD_HA_-AAV-sh*Nfia* mice, whisker stimulation-induced arteriolar dilation (Fig. 7H–J *p*< 0.001) and cerebral blood flow (Fig. 7K, L *p*< 0.01) were significantly reduced compared to the BHD_HA_-AAV-shNC group, reaching levels comparable to the Model-AAV-sh*Nfia* group. Barnes maze testing revealed that the escape latency ratio (Day 4/Day 1) in BHD_HA_-AAV-sh*Nfia* mice was significantly higher than in BHD_HA_-AAV-shNC mice (*p* < 0.05) (Fig. 7M, N). These results demonstrate that *Nfia* knockdown in SVZ cells abolishes BHD_HA_'s therapeutic effects on NVC function and cognitive recovery in CCI_QDBS_ model mice, suggesting that BHD_HA_ alleviates brain injury partially through NFIA upregulation, which promotes astrocyte development and restores neurovascular coupling.

## Discussion

The syndrome of QDBS is considered a key TCM syndrome for chronic cerebral ischemia [21]. Treating chronic cerebral ischemia by targeting QDBS syndrome has become a consensus in TCM, supported by a substantial amount of clinical and experimental data indicating that treatment targeting this syndrome can significantly improve neurological function in patients with cerebral ischemia [21, 33]. BHD is regarded as a fundamental prescription in TCM for treating QDBS after stroke [37]. Many traditional practitioners believe that the use of BHD for treating QDBS after stroke should emphasize the use of Astragalus, reasoning that increasing the dosage of Astragalus can enhance the Qi-tonifying effect, which in turn can promote blood circulation. However, there is currently no consensus on the dosage of Astragalus in the prescription, possibly due to the unclear importance and mechanisms of BHD_HA_, which greatly limits its clinical application. We combined the bilateral common carotid artery occlusion (BCCAO), systemic hypotension, and sleep deprivation (SD) to construct the CCI_QDBS_ model. In TCM theory, prolonged mental fatigue leads to 'Qi deficiency'. We utilized an SD apparatus to simulate chronic mental exhaustion, which is milder and more translatable to modern human lifestyles than the former extreme physical stress models (e.g., forced swimming), thus avoiding acute physical stress interference. Furthermore, BCCAO combined with temporary systemic hypotension effectively overcomes the individual anatomical variations in the mouse Circle of Willis, ensuring a uniform and stable cerebral hypoperfusion to mimic 'Blood stasis' caused by Qi deficiency. Our previous comprehensive evaluation confirmed that this model successfully replicates the clinical features of CCI_QDBS_, including declined muscle strength, dark purple tongue, and severe cognitive impairment [34]. In this study, we demonstrated that in a mouse model of CCI_QDBS,_ treatment with BHD_HA_ significantly improved physical signs such as tongue appearance, grip strength, and body weight. Furthermore, the treatment led to a notable recovery in cognitive performance and brain functional connectivity. Importantly, the therapeutic effect of BHD_HA_ was superior to that of BHD_LA_. These findings underscore the necessity of using high-dose Astragalus in BHD formulations to optimize therapeutic efficacy for CCI_QDBS_.

Next, this study explored the mechanisms by which BHD_HA_ exerts its therapeutic effects. NVC links neuronal activity to regional cerebral blood flow modulation, representing a core physiological mechanism of cerebral circulation. NVC dysfunction is closely implicated in diverse neurological disorders [28, 36]. Meanwhile, NVC activity lays the foundation for brain functional imaging signals [2]. We found that BHD_HA_ significantly altered brain functional connectivity in CCI_QDBS_ mice, prompting the hypothesis that BHD_HA_ may also influence NVC function. Previous studies have demonstrated that BHD can improve overall baseline cerebral blood flow, but investigations into its effects on the dynamic, activity-dependent regulation of blood flow via NVC remain unexplored [5]. Macroscopically, our global baseline hemodynamic mapping delineates the overall efficiency of the vascular network. This perspective not only functionally validates the successful establishment of our Qi deficiency and blood stasis model—evidenced by global sluggish circulation across all vessel diameters—but also demonstrates the holistic therapeutic effect of BHD_HA_ on general blood circulation (Fig. 3). Microscopically, specific quantitative comparisons of dynamic vascular reactivity in similar-sized microvessels further confirmed this mechanism. BHD_HA_ significantly improved the blood flow velocity of capillary vessels of the same diameter in the barrel cortex, the microvascular dilation capacity after whisker stimulation, and blood flow changes compared to the model group. Although BHD_LA_ could also increase baseline blood flow velocity, its ability to improve the regulation of blood flow in response to dynamic neural activity was significantly limited (Fig. 4). This suggests that the superior effect of BHD_HA_ compared to BHD_LA_ may be due to its ability to restore the regulation of NVC function. To elucidate its potential mechanisms, we conducted RNA-seq analysis and found that compared to the model group and the BHD_LA_ group, the BHD_HA_ significantly upregulated biological processes such as "astrocyte development", and "glial cell proliferation". Astrocytes constitute the most abundant cell population in the brain, and their perivascular endfeet wrap over 90% of cerebral capillaries, positioning astrocytes as pivotal mediators of NVC [14, 22, 27]. Astrocytes are ideally positioned to play a significant role in NVC. They detect neuronal activity and release vasoactive molecules such as PGE2 and EETs onto cerebral blood vessels via their end-feet, thereby altering vascular diameter to regulate blood flow [10, 20, 26]. This study proves that BHD_HA_ can mitigate the depletion of CAAs caused by the CCI_QDBS_ model, thereby enhancing NVC function. This beneficial effect is correlated with the upregulation of NFIA expression. Recent studies have revealed NFIA is critically important for the generation of reactive astrocytes following CNS injury [17]. Intriguingly, astrocytes induced by NFIA not only promote synaptogenesis but also exhibit neuroprotective properties [32]. Notably, the absence of NFIA in mature astrocytes triggers alterations in their morphology, molecular signatures, and synaptic support capabilities, ultimately leading to impaired synaptic plasticity and memory function [13]. Importantly, AAV-mediated knockdown of *Nfia* suppressed these beneficial effects of BHD_HA_, uncovering a novel therapeutic mechanism. However, the specific active ingredients in BHD_HA_ that regulate NFIA and astrocyte development have not yet been determined through research. Further exploration will benefit for fully elucidating the therapeutic advantages of BHD_HA_.

TCM considers Astragalus to be one of the most effective Qi-tonifying herbs. Our study validates the core TCM principle that high-dose Astragalus serves as the critical determinant of BHD's therapeutic efficacy in treating chronic cerebral ischemia associated QDBS. BHD_HA_ may exert Qi-tonifying and blood-activating effects by promoting astrocyte proliferation and strengthening their regulatory function in NVC.

## Conclusion

**Fig. 8:**
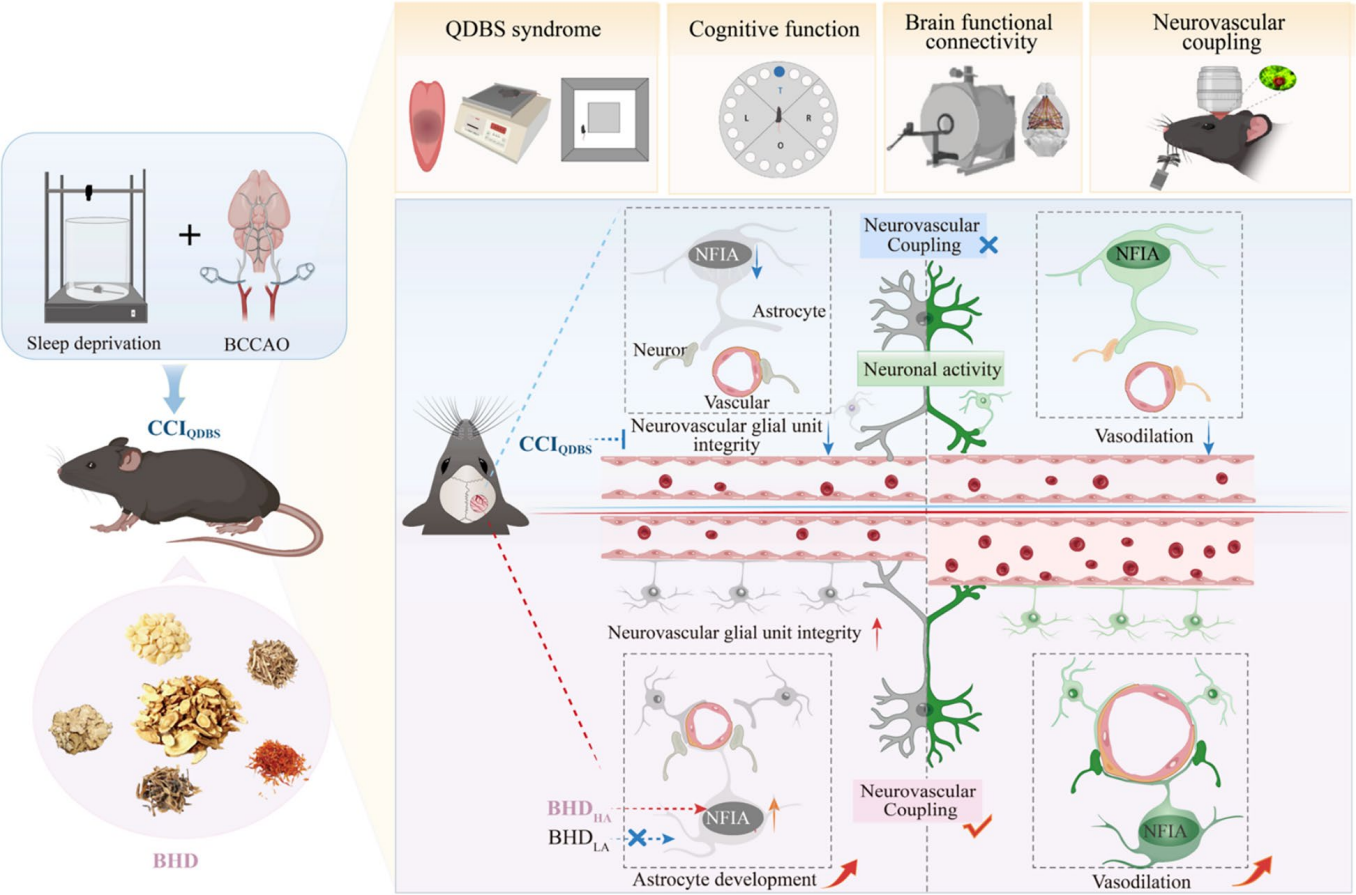
Schematic diagram illustrating that high-dose, but not low-dose Astragalus BHD maintains the stability of the neurovascular unit and improves neurovascular coupling by regulating *Nfia*. These mechanisms effectively reshape the brain's functional connectivity, leading to significant improvements in cognitive function and a reduction in symptoms associated with CCI_QDBS_

In summary, our study identifies high-dose Astragalus as critical for the therapeutic efficacy of BHD formulations under the conditions of the present experimental model. The superior therapeutic effects of BHD_HA_ likely arise through NFIA-mediated astrocyte replenishment, which maintains NVU stability, enhances NVC function, reshapes brain functional connectivity, and ultimately restores cognitive function (Fig. 8). This research provides a scientific rationale for the high-dose application of Astragalus in this classical TCM prescription, bridging traditional usage with modern mechanistic understanding.

## Supplementary Information

Supplementary Material 1

Supplementary Material 2

Supplementary Material 3

## Data Availability

The datasets used and/or analyzed during the current study are available from the corresponding author on reasonable request.

## Abbreviations

AAV: Adeno-associated virus
BCCAO: Bilateral common carotid artery occlusion with systemic hypotension
BHD: Buyang Huanwu Decoction
BHD_HA_: BHD with a high dose of Astragalus
BHD_LA_: BHD with a low dose of Astragalus
CAAs: Cerebrovascular-associated astrocytes
CCI: Chronic cerebral ischemia
CCI_QDBS_: Combined chronic cerebral ischemia with Qi Deficiency and Blood Stasis
DEGs: Differentially expressed genes
EETs: Epoxyeicosatrienoic acids
fMRI: Functional magnetic resonance imaging
Gin: Ginaton
NFIA: Nuclear factor IA
NVC: Neurovascular coupling
NVU: Neurovascular unit
PGE₂: Prostaglandin E₂
QDBS: Qi Deficiency and Blood Stasis
ROIs: Regions of interest
SVZ: Subventricular zone
TCM: Traditional Chinese medicine
