# Supplementary material for: Critical role of high-dose Astragalus in Buyang Huanwu Decoction for enhancing neurovascular coupling in a Qi Deficiency and Blood Stasis animal model
Source: Chin Med. 2026 Jun 18;21:170. doi: 10.1186/s13020-026-01443-8 (PMC13277118; doi:10.1186/s13020-026-01443-8)
Supplement: Supplementary file 3 — Supplementary Material 3 [file 13020_2026_1443_MOESM3_ESM.docx]

**Supplementary Information**


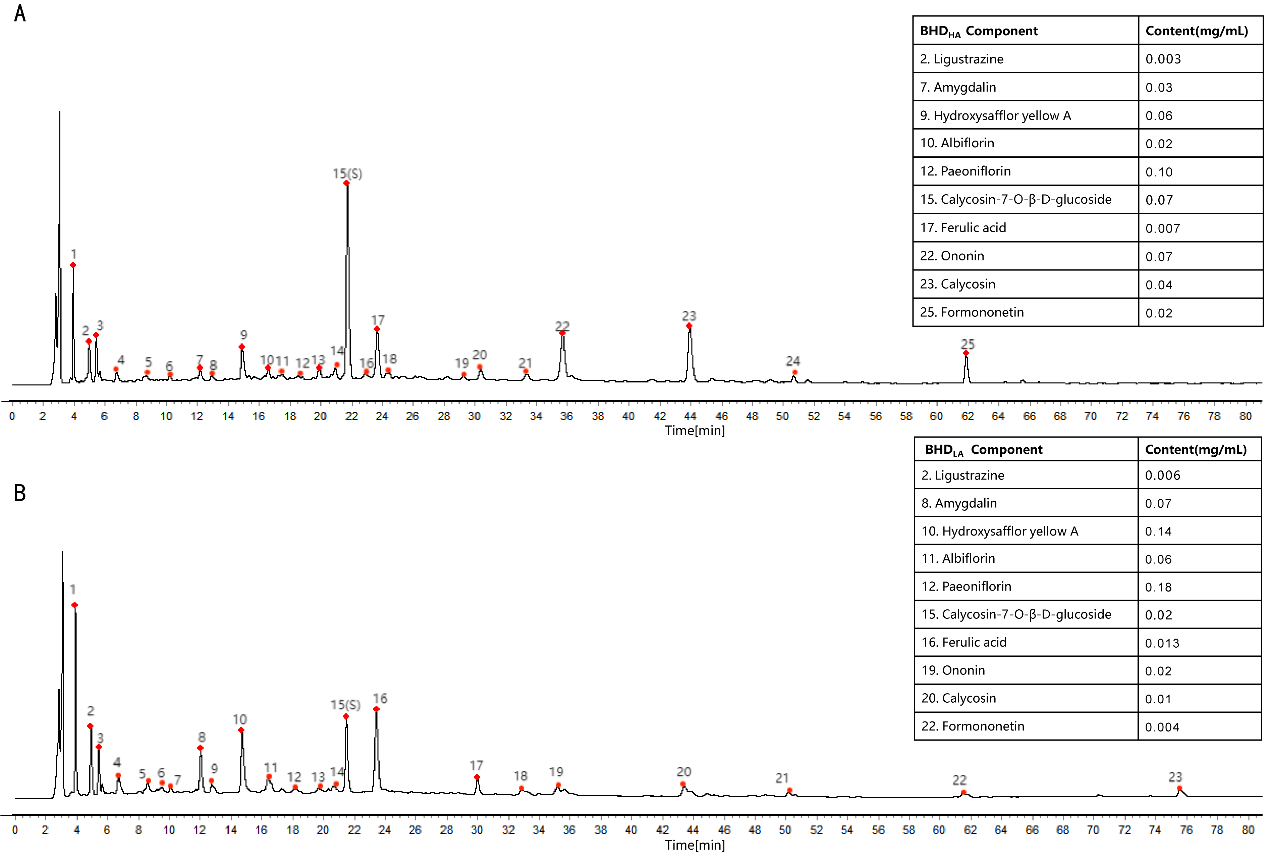


**Fig. S1 HPLC chromatograms and contents of main components of BHD with different dosages of Astragalus.**


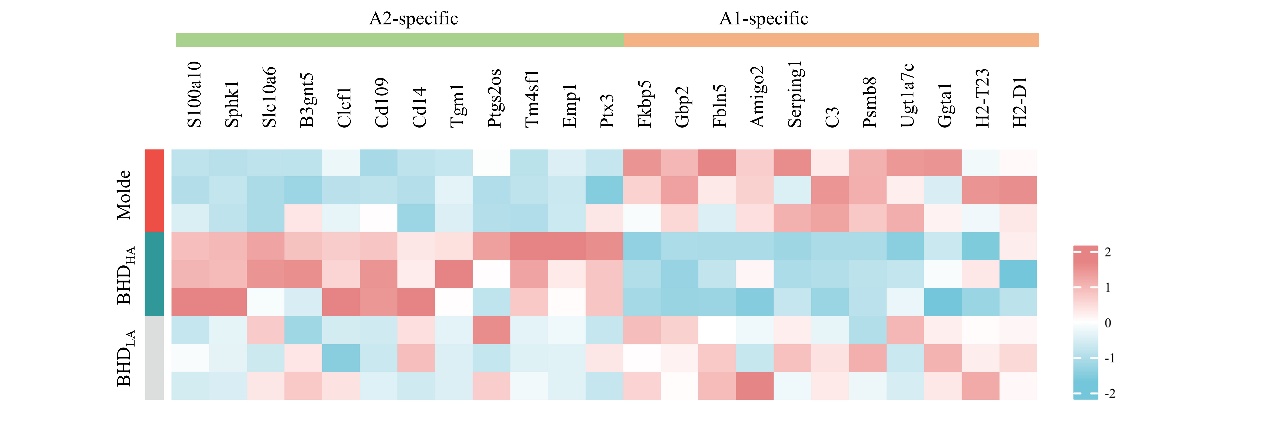


**Fig. S2 Heatmap of the relative mRNA levels of genes in A1-like astrocytes and A2-like astrocytes.** n=3.

**Movie. S1. Real-time NVC assessment in the mouse barrel cortex using two-photon microscopy.** The video displays neuronal activation (jGCaMP7s) and corresponding vascular responses (TRITC-Dextran) triggered by whisker stimulation.
